# Assessment of the Desire and Readiness of Taif Residents for Heart Donation After Death

**DOI:** 10.7759/cureus.36081

**Published:** 2023-03-13

**Authors:** Sultan M Alzahrani, Khalid Alzahrani, Moayyad Alotbi, Abdulrahman M Alamri, Rami F Algethami, Mohammed H Alfaqih, Hussam Alghamdi, Abdulaziz Al Rashed

**Affiliations:** 1 Internal Medicine, College of Medicine, Taif University, Taif, SAU; 2 Surgery, Taif University, Taif, SAU; 3 Medicine, Taif University, Taif, SAU; 4 Medicine and Surgery, Taif University, Taif, SAU

**Keywords:** saudi arabia, organ donation, desire, donation, heart transplantation

## Abstract

Background/aim

Heart transplantation is often the only preferable treatment for end-stage heart failure (HF); however, there are insufficient organ donors in Saudi Arabia. In this study, we aimed to understand the desire and readiness of Taif populations for heart donation after death.

Methods

We carried out a descriptive cross-sectional study among Taif residents in November 2022. A questionnaire designed from a previous survey was distributed among the participants. The questionnaire included sociodemographic data and questions assessing their desire for heart donation.

Results

The study included 405 subjects who have accepted to participate in the study. About half of the participants were aged 18 to 32 years (43.5%), most were females, were non-employed, and had a university degree. Of them, 86.2% accepted the concept of organ transplantation, 81% accepted the concept of heart transplantation, and one-third of the participants desired to donate their hearts. The participants with a university degree reported significantly less acceptance of the concept of heart transplantation (p-value=0.026), and those employed showed a significantly stronger desire for organ donation to a relative after death (p-value=0.049). In addition, younger participants showed a significantly higher willingness for organ donation to a relative or non-relative after death (p-value=0.017 and 0.009, respectively). Employed participants were significantly more willing to undergo heart transplantation surgery if needed (p-value=0.044).

Conclusion

Awareness campaigns could be established in the community and popularized during contact with the health system to build trust in the organ donation system, stress the importance of heart donation in saving the lives of more patients, and reduce the shortage of organ transplantation.

## Introduction

The prevalence of heart failure (HF) highly increases worldwide, especially among the aging population. In addition, the high prevalence of risk factors, such as ischemic heart disorder, diabetes, and obesity, correlated with growing HF incidence [1]. It was estimated that HF had affected about 26 million individuals worldwide, about 5% of whom, at the end stage, reported an extremely poor quality of life (QOL) [2]. The prevalence of HF in developed countries ranges from 1% to 2% and rises to 10% or more among those over 70 years [3].

Advanced HF therapies, such as durable mechanical circulatory support (MCS) and heart transplantation, could provide hope for improved survival rates and QOL [4]. Heart transplantation is preferable, as it significantly increases survival, exercise capacity, and return to work compared with conventional treatment [5]. In the last century, an increase in median survival post-transplantation from 11-18 days to 11 years has been achieved with the enhancement of antibiotics and immunotherapy [6].

Despite medical and technological improvements and high awareness of organ donation and transplantation importance, the gap between supply and demand continues to broaden, and the lack of organ donation has become a significant public health problem [7,8].

Heart transplantation has been considered the final treatment choice for patients suffering from end-stage HF in the last decade; however, it has stagnated due to the high demand and lack of donors [3,9]. Therefore, the shortage of heart donors is a significant cause of extended waitlist times and mortality [9].

The annual number of cardiac transplants has reached 5000 worldwide. In Saudi Arabia, there are only two cardiac centers that conduct a total of approximately 30 heart transplantations per year [10,11].

Recently, multiple attempts have been made to counter organ transplantation shortages such as reducing demand by improving preventive measures and increasing supply by increasing organ donation. One of the attempts to increase organ donation had been achieved by controlled donation after circulatory death (DCD) and brain death (DBD) cardiac allografts obtained from DCD donors have been encouraging compared to those obtained from DBD donors [9].

In Saudi Arabia, large efforts are provided to increase the donation process by enhancing awareness among different population classes [12]. Hence, we aimed to discuss the desire and readiness of the Taif population for heart donation after death to identify gaps between the demand for and availability of heart donors.

## Materials and methods

Study design and setting

This cross-sectional study was conducted among the population residing in Taif city, Saudi Arabia, in November 2022.

Study population

The study included subjects who lived in Taif city, Saudi Arabia and were willing to participate in the study. People who were working in the medical field were excluded. Verbal consent was taken from the participants, and the study proposal was approved by the ethical committee of Taif University with No. HAO-02-T-105.

Sample size

The sample size was calculated with a Roasoft sample size calculator (Raosoft, Inc., Seattle, WA), considering a 50% population proportion, a 95% confidence interval, and a 5% margin of error, and the minimum representative sample was 385 participants.

Data collection tool

The data collection tool was a questionnaire from a previous study in Saudi Arabia [3]. It consisted of two sections; a demographic section and a section of questions that assessed the participants’ desire and readiness toward heart donation after their death. The questionnaire was translated into Arabic to be easily understood by the participants.

Statistical analysis

After data extraction, they were revised and coded. The statistical calculations were done using the computer program IBM SPSS (version 26.0, Armonk, NY). Data were statistically described frequencies (number of cases), and valid percentages were used for categorical variables.

The chi-square test was used to determine the association between study questions and demographic variables. P-values less than 0.05 were considered statistically significant.

## Results

The study included 405 subjects who have accepted to participate in the study. Most of the participants were with a university degree (75.1%), were females (60%), and were non-employed (64%). Approximately half of the participants were aged 18 to 32 years and married (43.5% and 54.8%, respectively). Details pertaining to the respondent demographics are summarized in Table 1.

**Table 1:** Demographic characteristics of participants

Parameters	Category	Count (n=405)	Percentage
Age	Less than 18 years	5	1.2
	18-32 years	176	43.5
	33-50 years	143	35.3
	More than 50 years	81	20.0
Gender	Male	162	40.0
	Female	243	60.0
Marital status	Single	161	39.8
	Married	222	54.8
	Divorced	16	4.0
	Widowed	6	1.5
Educational level	Primary school	3	0.7
	Middle school	11	2.7
	Secondary	71	17.5
	University	304	75.1
	Postgraduate	16	4.0
Employment Status	Student	97	24.0
	Housewife	68	16.8
	Employee	146	36.0
	Unemployed	30	7.4
	Retired	64	15.8

According to questions that assessed the participants’ desire and readiness for organ and heart transplantations, 86.2% accepted the concept of organ transplantation and 13.8% of those refused it for religious reasons. In addition, most participants agreed with the humanity of organ transplantation and the concept of heart transplantation (95.3%, and 81%, respectively). Only one-third of the participants desired to donate their hearts, 67.9% agreed to donate their hearts to a relative after death, and 59.8% would donate their hearts to a non-relative after death.

Furthermore, when the respondents were asked if they prefer heart transplantation, 77.3% preferred heart transplantation for themselves. The details are described in Table 2.

**Table 2:** Respondents’ perspectives on organ transplantation and donation

	Response	Count (n= 405)	Percentage
Do you think the concept of organ transplantation is acceptable?	Yes	349	86.2
	No	56	13.8
If no, is it for religious reasons?	Yes	85	43.8
	No	109	56.2
Do you think organ transplantation is humane?	Yes	386	95.3
	No	19	4.7
Do you think the concept of heart transplantation is acceptable?	Yes	328	81.0
	No	77	19.0
If no, is it for religious reasons?	Yes	78	39.0
	No	122	61.0
Do you know someone who has had organ transplantation?	Yes	208	51.4
	No	197	48.6
Do you know someone who has had a heart transplant?	Yes	48	11.9
	No	357	88.1
Do you know someone who is waiting for an organ transplant?	Yes	130	32.1
	No	275	67.9
Do you think organ donation saves lives?	Yes	391	96.5
	No	14	3.5
Do you think organ donation is regulated?	Yes	321	79.3
	No	84	20.7
Do you have a heart problem?	Yes	21	5.2
	No	384	94.8
Do you have a relative with a heart problem?	Yes	237	58.5
	No	168	41.5
Have you ever donated blood?	Yes	137	33.8
	No	268	66.2
Would you donate an organ while alive to a relative?	Yes	256	63.2
	No	149	36.8
Would you donate an organ to a non-relative whilst alive?	Yes	124	30.6
	No	281	69.4
Would you donate a heart?	Yes	121	29.9
	No	284	70.1
Would you donate an organ to a relative after your death?	Yes	275	67.9
	No	130	32.1
Would you donate an organ to a non-relative after your death?	Yes	242	59.8
	No	163	40.2
Would you consent to the organ donation of a relative whilst they are alive?	Yes	159	39.3
	No	246	60.7
Do you think doctors would try less to save your life if they knew that you were a registered organ donor?	Yes	165	40.7
	No	240	59.3
Do you think that the body is treated respectfully by doctors and nurses after organ donation?	Yes	326	80.5
	No	79	19.5
Do you think the heart is taken from a person after death?	Yes	245	60.5
	No	160	39.5
Do you think the heart is taken whilst the person is alive?	Yes	130	32.1
	No	275	67.9
Have you heard of brain death?	Yes	395	97.5
	No	10	2.5
Do you know what brain death means?	Yes	361	89.1
	No	44	10.9
Have you heard of artificial hearts?	Yes	247	61.0
	No	158	39.0
If needed for yourself, would you prefer heart transplantation?	Yes	313	77.3
	No	92	22.7
If needed for yourself, would you prefer an artificial heart?	Yes	244	60.2
	No	161	39.8
If needed for a family member, would you prefer an artificial heart?	Yes	281	69.4
	No	124	30.6

Unfortunately, 53.3% of the total participants who accepted the heart transplantation concept refused to donate a heart, whereas one-third (27.6%) of them accepted the heart concept and to donate a heart. In addition, 16.8% did not accept the concept of heart transplantation and donation.

It was found that more than half of the participants (54.5%) who did not accept the heart transplantation concept accepted to undergo heart transplantation surgery if needed, and 17.4% accepted the heart transplantation concept and refused to undergo the surgery.

The independent factors were compared with the participant’s acceptance of the organ transplantation concept, and there was no significant impact of these factors on the acceptance of the organ transplantation concept (Table 3).

**Table 3:** Perspectives on the acceptance of the organ transplantation concept among the different subgroups

Factors		Do you think the concept of organ transplantation is acceptable?		Total (n=405)	P-value
		Yes (n=349)	No (n=56)		
Sex	Male	137 (84.6)	25 (15.4)	162	0.445
	Female	212 (87.2)	31(12.8)	243	
Education	With no university degree	77 (90.6)	8 (9.4)	85	0.185
	Had a university degree	272 (85.0)	48 (15.0)	320	
Age	Less than 33 years old	150 (82.9)	31(17.1)	181	0.084
	33 years or older	199 (88.8)	25 (11.2)	224	
Marital status	Unmarried	153 (83.6)	30 (16.4)	183	0.174
	Married	196 (88.3)	26 (11.7)	222	
Employment status	Unemployed	218 (84.2)	41 (15.8)	259	0.120
	Employed	131 (89.7)	15 (10.3)	146	

Moreover, in comparing the demographic characteristics with the acceptance of the heart transplantation concept, the participants with a university degree reported significantly less acceptance of the concept of heart transplantation (78.8%, and 89.4%, respectively) (p-value=0.026). All details are illustrated in Table 4.

**Table 4:** Perspectives on acceptance of the heart transplantation concept among the different subgroups

Factors		Do you think the concept of heart transplantation is acceptable?		Total (n=405)	P-value
		Yes (n= 328)	No (n=77)		
Sex	Male	127 (78.4)	35 (21.6)	162	0.278
	Female	201 (82.7)	42 (17.3)	243	
Education	With no university degree	76 (89.4)	9 (10.6)	85	0.026*
	Had a university degree	252 (78.8)	68 (21.3)	320	
Age	Less than 33 years old	141 (77.9)	40 (22.1)	181	0.155
	33 years or older	187 (83.5)	37 (16.5)	224	
Marital status	Unmarried	142 (77.6)	41 (22.4)	183	0.114
	Married	186 (83.8)	36 (16.2)	222	
Employment status	Unemployed	203 (78.4)	56 (21.6)	259	0.075
	Employed	125 (85.6)	21 (14.4)	146	

In addition, all demographic characteristics did not significantly affect the participants' willingness to donate their hearts (Table 5).

**Table 5:** Perspectives on organ donation among the different subgroups

Factors		Would you donate a heart?		Total (n=405)	P-value
		Yes (n=121)	No (n=284)		
Sex	Male	44 (27.2)	118 (72.8)	162	0.330
	Female	77 (31.7)	166 (68.3)	243	
Education	With no university degree	29 (34.1)	56 (65.9)	85	0.337
	Had a university degree	92 (28.7)	228 (71.3)	320	
Age	Less than 33 years old	60 (33.1)	121 (66.9)	181	0.196
	33 years or older	61 (27.2)	163 (72.8)	224	
Marital status	Unmarried	60 (32.8)	123 (67.2)	183	0.245
	Married	61 (27.5)	161 (72.5)	222	
Employment status	Unemployed	79 (30.5)	180 (69.5)	259	0.714
	Employed	42 (28.8)	104 (71.2)	146	

The factors were also compared with the participants’ desire for organ donation to a relative person after his/her death. The age and employment status significantly affected their willingness to organ donation to a relative after death. Employed participants had a significantly stronger desire for organ donation to a relative after death (74%) than unemployed participants (64.5%) (p-value= 0.049). In addition, those aged 33 years or less showed a significantly stronger desire toward organ donation to a relative after death (74%) than older participants (62.9%) (p-value=0.017). All information is described in Table 6.

**Table 6:** Willingness for heart donation to a relative among the different subgroups

Factors		Would you donate an organ to a relative after your death?		Total (n=405)	P-value
		Yes (n=275)	No (n=130)		
Sex	Male	110 (67.9)	52 (32.1)	162	1.00
	Female	165 (67.9)	78 (32.1)	243	
Education	With no university degree	58 (68.2)	27 (31.8)	85	0.941
	Had a university degree	217 (67.8)	103 (32.3)	320	
Age	Less than 33 years old	134 (74)	47 (26)	181	0.017*
	33 years or older	141 (62.9)	83 (37.1)	224	
Marital status	Unmarried	131 (71.6)	52 (28.4)	183	0.149
	Married	144 (64.9)	78 (35.1)	222	
Employment status	Unemployed	167 (64.5)	92 (35.5)	259	0.049*
	Employed	108 (74)	38 (26)	146	

Regarding willingness to donate a heart to a non-relative person, individuals who are 33 years or older were less likely to donate their organs to a non-relative person after death (54%) than younger participants (66.9%) (p-value=0.009) (Table 7).

**Table 7:** Willingness toward heart donation to a non-relative among the different subgroups

Factors		Would you donate an organ to a non-relative whilst alive?		Total (n=405)	P-value
		Yes (n=242)	No (n=263)		
Sex	Male	91 (56.2)	71 (43.8)	162	0.230
	Female	151 (62.1)	92 (37.9)	243	
Education	With no university degree	54 (63.5)	31 (36.5)	85	0.424
	Had a university degree	188 (58.8)	132 (41.3)	320	
Age	Less than 33 years old	121 (66.9)	60 (33.1)	181	0.009*
	33 years or older	121 (54)	103 (46)	224	
Marital status	Unmarried	117 (63.9)	66 (36.1)	183	0.119
	Married	125 (56.3)	97 (43.7)	222	
Employment status	Unemployed	154 (59.5)	105 (40.5)	259	0.872
	Employed	88 (60.3)	58 (39.7)	146	

With respect to desiring heart transplantation, employed participants were significantly more willing to undergo heart transplantation surgery if needed (82.9%) than those unemployed (74.1%) (p-value=0.044). The details are illustrated in Table 8.

**Table 8:** Participants’ desire to undergo heart transplantation among the different subgroups

Factors		If needed for yourself, would you prefer heart transplantation?		Total (n=405)	P-value
		Yes (n= 313)	No (n=92)		
Sex	Male	130 (80.2)	32 (19.8)	162	0.245
	Female	183 (75.3)	60 (24.7)	243	
Education	With no university degree	69 (81.2)	16 (18.8)	85	0.335
	Had a university degree	244 (76.3)	76 (23.8)	320	
Age	Less than 33 years old	140 (77.3)	41 (22.7)	181	0.978
	33 years or older	173 (77.2)	51 (22.8)	224	
Marital status	Unmarried	138 (75.4)	45 (24.6)	183	0.414
	Married	175 (78.8)	47 (21.2)	222	
Employment status	Unemployed	192 (74.1)	67 (25.9)	259	0.044*
	Employed	121(82.9)	25 (17.1)	146	

## Discussion

Heart transplantation has been globally accepted as the best treatment option for patients suffering from end-stage HF [13]. On the other hand, there is an increase in wait lists at rates higher than transplant rates; thus, there is an urgent need to encourage organ transplantation to limit the organ supply shortage and save more patients' lives [9]. In this study, we aimed to assess the desire and readiness of Taif city, Saudi Arabia’s population for heart donation after death.

Literature reviews reported that organ donation is not common in Saudi Arabia, and some studies have suggested that this is caused by social stigma and lack of information [14]. However, in the present study, a high number of the participants accepted organ and heart transplant concepts. According to a previous study in Saudi Arabia, results were consistent with ours in accepting the organ and heart transplant concept. [3]. Another study in Hong Kong reported that 85.2% of the respondents supported organ transplants [15]. Additionally, in an Indian study, the organ donation concept became familiar and acceptable among the general population [16].

There is variability in the impact of educational level on the acceptance of organ and heart donation concepts. In the present study, it was surprising that participants with lower education had significantly higher acceptance of the organ donation concept. The education level did not impact the acceptance of the heart donation concept. Our results were inconsistent with the fact that education could increase the public's awareness of the importance of organ donation [17]. In addition, previous studies in Saudi Arabia indicated that education level did not impact the acceptance of organ or heart transplantation concepts [3,12].

Several beliefs, including knowledge and religious beliefs, could play a role in determining a person’s view of organ donation [18]. It was found that about half of the participants refused the heart and organ concepts due to religious barriers. These results are similar to a study conducted in Jazan, Saudi Arabia, which reported that 39% of participants considered religion a barrier to organ donation [12]. Another study in Saudi Arabia reported a close percentage of 42.4% of heart transplantation rejection for religious reasons [3].

Most participants thought organ donation saves lives; however, many were unwilling to donate a heart. Furthermore, it was found that a relatively high percentage (65.9%) of respondents who have accepted the heart transplantation concept refused to donate their hearts. Their refusal of heart donation might have resulted from the fact that approximately half of them did not trust that doctors would make an effort to save their life if they registered as organ donors. Additionally, the National Health Services Blood and Transplant reported that the most common issue regarding organ donation was a fear that medical staff may not do their best to save lives if the patient is a registered donor [19]. Therefore, it is possible that establishing regulations that guarantee the donors better medical care and easy access to health facilities would encourage people to donate organs during their lifetimes [17].

According to a study in Belgium, the acceptance of organ donation reduced with increasing age from 85.7% in young adults to 63.6% among grandparents [20]. In addition, a previous Saudi study reported that the younger generation was more accepting of the change and willing to donate an organ [3]. In the same concept, younger participants in our study showed a higher willingness to donate an organ to relatives and non-relative persons. These results may focus on younger generations being more open-minded about new concepts.

One-third of the participants in this study had not heard of artificial hearts; thus, when they were given a choice between heart transplantation and artificial hearts for themselves, they preferred heart transplantation. Hence, the role of physicians and healthcare providers is essential to provide more information about all treatment aspects of HF.

In this study, about half of the participants who refused the concept of heart transplantation would undergo heart transplantation surgery if needed. This conflict may arise from the lack of empathy toward others and could indicate that people may change their thoughts according to the disorder’s severity and save their lives. It also may give a key to encouraging stakeholders to introduce sufficient information about the importance of heart transplantation for the survival of patients.

Limitations

This study assessed the participants’ desire for heart and organ donation without identifying the gaps in their knowledge about the donation concepts. Further studies should determine the knowledge level of the participants and its reflection on their desires.

## Conclusions

Heart donation is voluntary and no one should be blamed for not wanting to participate in it. In this study, almost all the participants considered organ donation humane, and many accepted the heart transplantation concept. Still, only a few had the desire to donate their heart. Awareness campaigns could be established in the community and popularized during contact with the health system to build trust in the organ donation system, increase awareness of the benefits of DBD to the patients, encourage the importance of heart donation in saving more patients' lives, and reduce the shortage in organ donation transplantation.
